# Value of temporal muscle thickness in predicting outcomes of stroke: a systematic review and meta-analysis

**DOI:** 10.3389/fneur.2025.1639425

**Published:** 2025-08-07

**Authors:** Min Fang, Fangjun Wang, Yu Sheng, Shengchen Qiu

**Affiliations:** ^1^Department of Nursing, The Fourth Affiliated Hospital of School of Medicine, International School of Medicine, International Institutes of Medicine, Zhejiang University, Yiwu, China; ^2^Department of Rehabilitation Medicine, The Fourth Affiliated Hospital of School of Medicine, International School of Medicine, International Institutes of Medicine, Zhejiang University, Yiwu, China

**Keywords:** sarcopenia, stroke, skeletal muscle thickness, mortality, dysphagia, functional outcomes

## Abstract

**Objective:**

This systematic review examined if temporal muscle thickness (TMT) as a surrogate marker of sarcopenia was a predictor of outcomes after stroke.

**Methods:**

We explored the PubMed, Embase, Scopus, and Web of Science databases till 18th January 2025 for studies reporting stroke prognosis based on baseline TMT. Pooled analyses examined associations with post-stroke mortality, functional outcomes, and dysphagia. Meta-regression was conducted using baseline NIHSS and TMT values as moderators.

**Results:**

Nine studies were available. Meta-analysis showed that low TMT was associated with a statistically significant increase in the risk of post-stroke mortality (OR: 1.42 95% CI: 1.03, 1.96 *I*^2^ = 43%). A pooled analysis of five studies showed that there was a tendency for good functional outcomes with high TMT (OR: 1.24 95% CI: 1.00, 1.54 *I*^2^ = 75%). But the results were non-significant. Two studies showed that baseline TMT was significantly associated with an increased risk of dysphagia. Meta-regression did not identify significant moderating effects.

**Conclusion:**

Preliminary evidence suggests that lower TMT is associated with higher mortality after stroke, while associations with functional recovery remain inconclusive. Current limitations, including heterogeneity and lack of sex-specific analyses, preclude routine clinical use. Further standardized and patient-level research is warranted.

**Systematic review registration:**

https://www.crd.york.ac.uk/prospero/, identifier CRD42025637925.

## Introduction

Stroke is among the commonest cerebrovascular disease characterized by abrupt impairment in brain function usually caused by intra-cerebral hemorrhage or ischemia. It is the second most common cause of death worldwide and a major cause of prolonged disability (1). Nearly 16.9 million individuals are diagnosed with stroke every year with the majority being ischemic in etiology (2). Since about three of every four cases occur in the elderly (3), there has been a trend of increased prevalence of stroke with prolonged life expectancy (4). The geriatric age group is particularly at a higher risk of adverse outcomes like neurological deficits, disability, and risk of falls and fractures (5). Estimates show that octogenarians have a three-fold higher risk of short-term fatality after stroke making them a highly vulnerable population (6).

One of the causes of adverse stroke outcomes in the elderly is sarcopenia which is characterized by age-related depletion of skeletal muscle mass and strength (7). This involuntary muscle loss may begin as early as the fourth decade of life progressing linearly to deplete about 50% of muscle mass by the eighth decade (8). Importantly, sarcopenia is not caused only by aging but malnutrition, physical inactivity, and other diseases like bone diseases, and neurological disorders are also leading contributors (9). In view of the proven adverse effects of sarcopenia in stroke, the need for quantifying sarcopenia cannot be underestimated. However, there remains uncertainty on a consensus clinical definition of sarcopenia due to the lack of standardized measurements that record functional skeletal muscle mass (10, 11). Nevertheless, given the difficulties in measuring sarcopenia and the need for its routine quantification in clinical practice, the European Working Group on Sarcopenia in Older People (EWGSOP) has suggested that measures of low muscle mass and low muscle strength or performance could be used as a clinical definition (12). Computed tomography (CT) or magnetic resonance imaging (MRI) based quantification of skeletal muscle mass or functional measures like handgrip strength, knee flexion/extension, the short physical performance battery, gait speed, and timed up and go, etc. are being used to assess sarcopenia in routine practice (10, 11). In an acute stroke setting, most of these measures become redundant as the patient may be paralyzed or have cognitive disturbances.

In this regard, temporal muscle thickness (TMT) has been reported as a novel surrogate marker of sarcopenia (13, 14). Stroke patients usually undergo a brain CT immediately on admission and TMT can be easily measured in such patients without any additional investigations (14). Its use has been widely reported in prognosticating patients with brain tumors (13), however, its utility in stroke remains underexplored. In the past few years, a number of studies have been conducted assessing the role of TMT in prognosticating stroke patients, however, results have varied and there is limited clarity if TMT can be used in routine clinical practice to predict prognosis in stroke patients. Since, there has been no collated evidence to date, we conducted this systematic review to examine if the TMT could be used as a predictor for assessing outcomes in stroke patients.

## Methods

We conducted this systematic review and meta-analysis in accordance with PRISMA guidelines (15). The protocol of the review can be accessed on PROSPERO (CRD42025637925).

To answer the review question, we aimed to include all published articles in peer-reviewed journals and carried out on adult acute ischemic or hemorrhagic stroke patients. Studies were to report stroke outcomes based on baseline CT-measured TMT. All outcomes were acceptable for inclusion in the study. Outcomes were to be reported either as raw data or odds ratio (OR) with 95% confidence intervals (CI). We did not include conference abstracts, reviews, or case reports. Studies with overlapping data were to be excluded and the article with a maximum sample size from a particular database was to be included.

Literature was searched by two reviewers on the websites of PubMed, Embase, Scopus, and Web of Science. We approached a medical librarian to help formulate the search strategy which was to be used across all mentioned databases. It was: (((((((sarcopenia) OR (muscle wasting)) OR (skeletal muscle)) OR (age-related muscle loss)) OR (myopenia)) OR (cachexia)) AND ((((stroke) OR (cerebrovascular accident)) OR (intracerebral hemorrhage)) OR (cerebral ischemia))) AND (temporal muscle). We completed the search on 18th January 2025. No restrictions were placed on location, publication time, or language. The database search was also supplemented by hand-search of the reference list of included articles. Google Scholar was searched for gray literature. Studies searched on the databases were screened for appropriateness to the review by two authors by abstract reading. Relevant articles were further evaluated by downloading the full-texts which was also done by the same two authors. If there were disagreements, these were solved by discussion and consensus.

We prepared a table to obtain data from the studies. Details extracted were: first author, year of publication, design, type of stroke, demographic details, baseline NIHSS score, comorbidities, prior stroke history, TMT measurement level, TMT cut-off, and outcomes reported. We emailed the corresponding author once if the outcome was not reported in exact numbers allowing a meta-analysis.

The quality of the included literature was examined by the Newcastle Ottawa scale (NOS) (16). The scale determines bias in the article for selection of cohort, comparability of groups, and outcome assessment. Two authors judged each study for these domains and gave them scores ranging from zero to nine, the latter indicating high quality.

Data analysis was conducted on “Review Manager” (RevMan, version 5.3). All outcome data was tabulated and if a minimum of three studies were available for an outcome, a pooled analysis was conducted. For other outcomes, only a descriptive analysis was conducted. Post-stroke mortality was considered as the primary outcome while good functional outcomes and any other outcomes reported were considered as secondary outcomes. Data was pooled as OR and 95% CI in a random-effects model. Heterogeneity among studies was assessed through the *I^2^* index. *I^2^* of over 50% and/or *p* < 0.05 indicated substantial heterogeneity. Leave-on-out analysis function of the software was used to assess the impact of each study on the pooled analysis. As the number of included studies was <10, publication bias was not checked using funnel plots. Meta-regression was conducted using Comprehensive meta-analysis software (Version 3) for mortality and good functional outcomes using the moderators TMT thickness, and baseline NIHSS score.

## Results

### Search, baseline details, and study quality

**Figure 1:**
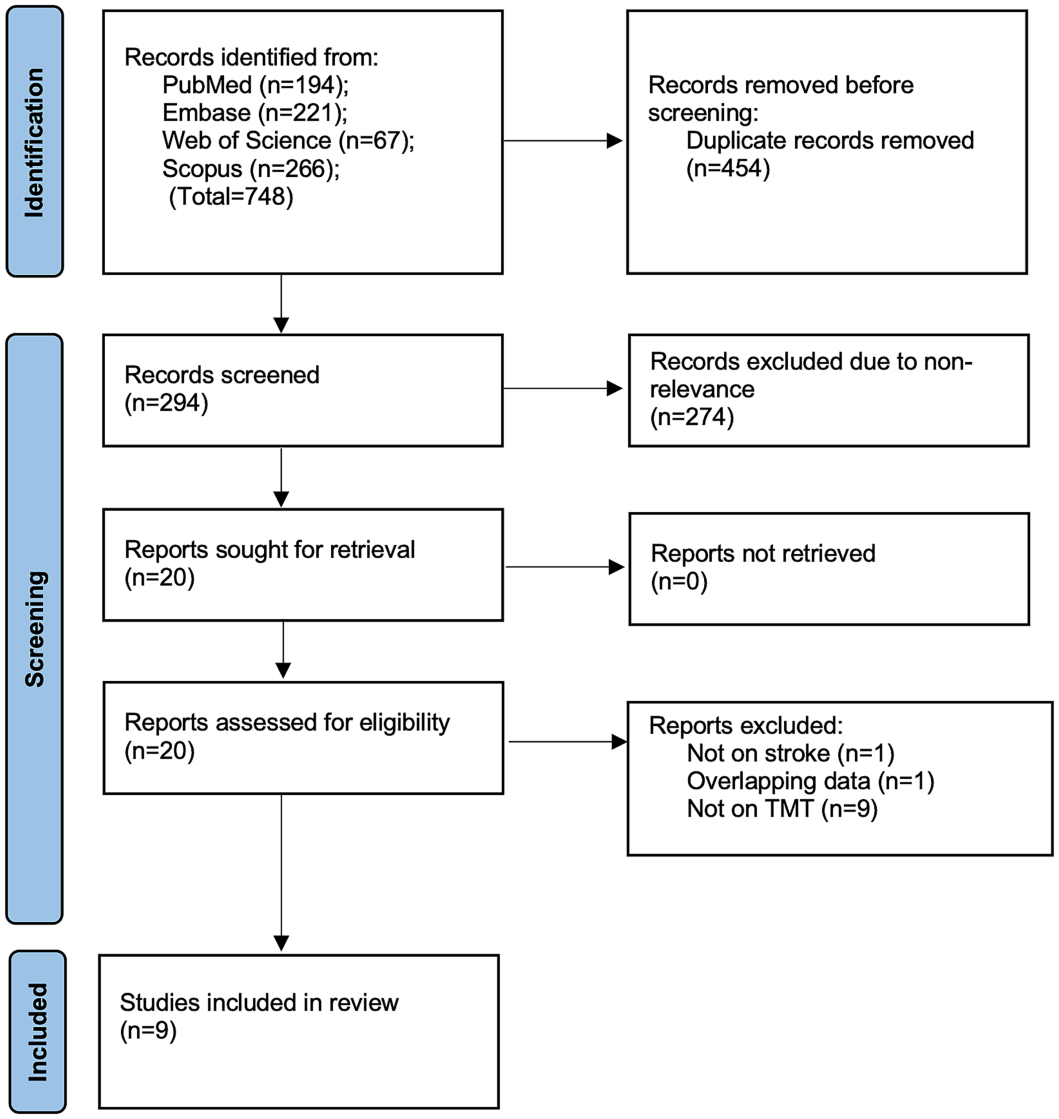
Study flowchart.

The database search led to 748 articles. After eliminating duplicates and non-relevant studies, 20 articles were selected for full-text analysis. In total, nine studies were found to be eligible for inclusion (17–25) (Figure 1).

The included articles were published between 2021 and 2024 presenting data from Japan, China, Taiwan, Turkey, Italy, and Germany. All had a similar cohort design, six included only ischemic stroke, and three included both ischemic and hemorrhagic stroke. The minimum sample size was 70 while the maximum was 657. A majority had patients with a mean age of >70 years. The baseline NHISS score varied widely indicating significant variation in the severity of stroke in the included studies. TMT was commonly measured at the level of the orbital roof. All studies used the baseline CT conducted for diagnosis. Four studies reported data on mortality, six reported data on good functional outcomes, and two reported data on post-stroke dysphagia.

On examination of the quality of studies, all studies received full points for selection of cohort. However, the two points for comparability of cohorts (high and low TMT) were not awarded to all studies as no study conducted baseline matching of high and low TMT groups. For outcome assessment, a maximum of three points could be awarded to any study and all studies received two or three points. The total NOS score therefore varied from six to seven (Table 1).

**Table 1:** Information retrieved from the included studies.

Study ID	Location	Design	Stroke type	Sample size	Age	Males (%)	NIHSS score	DM (%)	HT (%)	Prior stoke (%)	AF (%)	TMT level	Mean TMT (mm)	TMT cut-off (mm)	Outcomes	NOS score
Nozoe 2021	Japan	Cohort	Both	289	76	56.4	3 ± 4	24	50	26	8	Orbital roof	5.55 ± 1.69	4.6	Functional outcomes at 3 months (mRS)	7
Sakai 2021	Japan	Cohort	Both	70	75.6	61.4	10[7–18]	31.4	60	NR	NR	Orbital roof	5.45 ± 1.55	NR	Dysphagia	6
Li 2022	China	Cohort	Ischemic	265	NR	62.6	>5: 25.7%	33.2	82.6	NR	NR	Orbital roof	NR	NR	Mortality	7
Dubinski 2023	Germany	Cohort	Ischemic	282	71	45	5[2–6]	27	79	NR	24	NR	5[0–7]		Functional outcomes at discharge and 12 months (mRS)	7
Gursoy 2023	Turkey	Cohort	Ischemic	147	67.6	72	15.9 ± 4.3	37	88	NR	32	Orbital roof	5.6 ± 1.3	5.2	Mortality, Functional outcomes at 3 months (mRS)	7
Lin 2023	Taiwan	Cohort	Ischemic	657	72	52.1	18[13–23]	33.8	70.6	25.7	58.8	Orbital roof	6.35 ± 1.84	NR	Mortality, Functional outcomes at 3 months (mRS), sICH, any hemorrhage; early neurological improvement	7
Yang 2023	Taiwan	Cohort	Ischemic	148	73.1	45.9	17.6[14–22]	37.8	72.3	NR	58.1	Orbital roof	5.9 ± 1.6	7.2	Dysphagia	6
Ongun 2024	Turkey	Cohort	Both	114	78.6	51.8	10.2 ± 5.5	24.5	47.3	NR	14.9	Orbital roof	5.85 ± 0.96	NR	Functional outcomes at 3 months (mRS)	6
Ravera 2024	Italy	Cohort	Ischemic	291	73	54	11 [12]	19	70	10	25	Orbital roof	5.2 ± 1.9	Male: 6.3 Females: 5.2	Mortality, Functional outcomes at 3 months (mRS)	7

mRS, modified Ranking scale; NR, not reported; TMT, temporal muscle thickness; NOS, Newcastle Ottawa scale; DM, diabetes mellitus; HT, hypertension; AF, atrial fibrillation; sICH, symptomatic intracerebral hemorrhage.

### Outcomes

Detailed results of each study along with all reported outcomes are presented in Table 2. We could only assess mortality, functional outcomes, and dysphagia as it was reported by more than one study.

**Table 2:** Detailed representation of outcomes reported by included studies.

Study ID	Outcome	Results
Nozoe 2021	Functional outcomes at 3 months	Risk of poor functional outcomes was not significantly different between high and low TMT groups. Mean temporal muscle thickness was not significantly different between good or poor functional outcome (5.64 ± 1.64 mm vs. 5.30 ± 1.81 mm, *p* = 0.128).
Sakai 2021	Dysphagia	The median TMT values were 4.70, 5.75, and 5.75 mm in the severe dysphagia, mild dysphagia, and normal groups, respectively. TMT was found to be significant predictor of dysphagia severity following stroke (*p* = 0.036).
Li 2022	Mortality	TMT was significantly related to mortality (area under curve, 0.83; 95% CI, 0.76–0.90; *p* < 0.001). TMT was a significant predictor of mortality (*p* < 0.01).
Dubinski 2023	Functional outcomes at discharge and 12 months	A statistical significant association was noted between low TMT, reduced NIHSS and mRS at discharge (*p* = 0.035, *p* = 0.004), and reduced mRS at 12 months (*p* = 0.001).
Gursoy 2023	Mortality	A significant cutoff of ≤5.20 was determined for TMT in estimating mortality (AUC = 0.636, *p* = 0.012), which provided a sensitivity of 65%, specificity of 65%, positive predictive value of 42.62%, and negative predictive value of 82.28%. On multivariate analysis, no statistical significant association was noted between TMT and mortality (*p* = 0.53).
	Functional outcomes at 3 months	No significant cut-off was noted for TMT to predict functional outcomes. On multivariate analysis, no statistical significant association was noted between TMT and functional outcomes (*p* = 0.8).
Lin 2023	Mortality	No statistical significant association noted between TMT and mortality (*p* = 0.15)
	Functional outcomes at 3 months	Statistical significant association noted between high TMT and good functional outcomes (*p* = 0.004)
	sICH	No statistical significant association noted between TMT and sICH (*p* = 0.64)
	Any hemorrhage	No statistical significant association noted between TMT and sICH (*p* = 0.63)
	Early neurological improvement	No statistical significant association noted between TMT and early neurological improvement (*p* = 0.65)
Yang 2023	Dysphagia at 4 and 12 weeks	Statistical significant association noted between TMT and dysphagia at 4 weeks (p < 0.001) and 12 weeks (*p* < 0.001).
Ongun 2024	Functional outcomes at 3 months	A significant correlation was found between TMT on admission and the third month mRS score (*r* = −0.613, *p* < 0.001)
Ravera 2024	Mortality	Higher TMT values were independently associated with a decreased mortality risk (*p* = 0.01)
	Functional outcomes at 3 months	No statistical significant association noted between TMT and functional outcomes (*p* = 0.22)

CI, confidence interval; TMT, temporal muscle thickness; mRS, modified ranking scale; NIHSS, National Institutes of Health Stroke Scale; AUC, area under the curve; sICH, symptomatic intracerebral hemorrhage.

**Figure 2:**
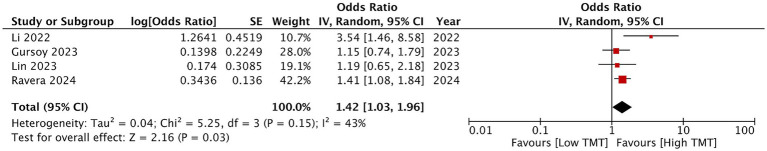
Pooled analysis for the association between TMT and mortality.

**Figure 3:**
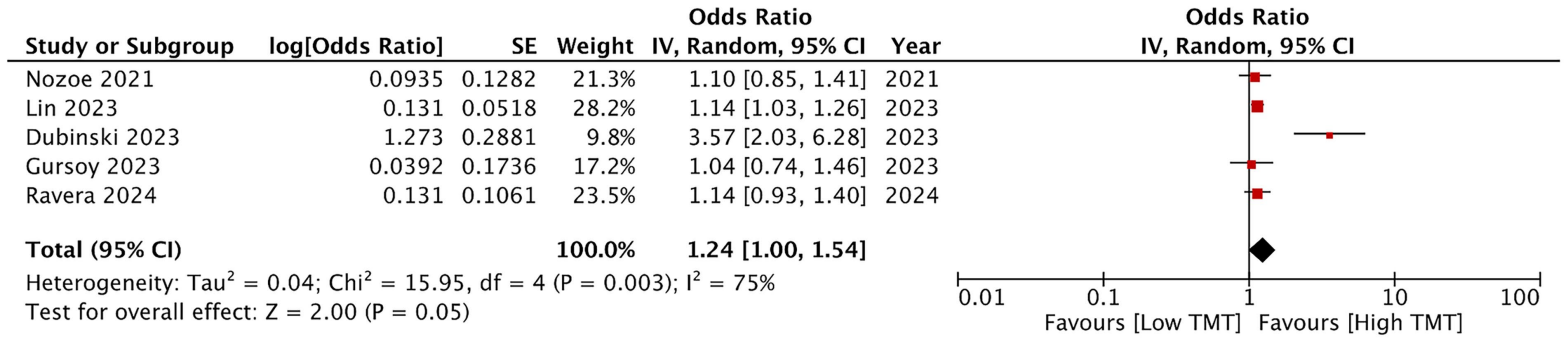
Pooled analysis for the association between TMT and good functional outcomes.

Analyzing mortality data, we noted that low TMT was associated with a statistically significant increase in the risk of post-stroke mortality (OR: 1.42 95% CI: 1.03, 1.96 *I*^2^ = 43%) (Figure 2). On sensitivity analysis, it was seen that the study of Ravera et al. (25) influences the overall results. The exclusion of this study led to a non-significant association between TMT and mortality (OR: 1.52 95% CI: 0.85, 2.72 *I*^2^ = 62%). Pooled analysis of five studies showed that there was a tendency for good functional outcomes with high TMT (OR: 1.24 95% CI: 1.00, 1.54 *I*^2^ = 75%) as the *p*-value was 0.05 (Figure 3). Good functional outcomes were defined as a modified ranking scale (mRS) score of 0–2 in most studies. The study of Ravera et al. (25) defined it as mRS of 0–3. The results of this analysis were not robust on sensitivity analysis turning significant on the exclusion of Gursoy et al. (21) and Dubinski et al. (20) and non-significant on the exclusion of the remaining studies. One study by Ongun et al. (24) only reported correlation data between TMT and functional outcomes and did not report either raw data or ORs. The authors found that there was a statistically significant correlation between mean TMT and mRS score at 3 months (*r* = −0.613, *p* < 0.001).

Meta-regression analysis was conducted for both mortality and good functional outcomes. We noted that the moderators, baseline TMT thickness and NIHSS score did not significantly influence the effect size of both outcomes (Table 3).

**Table 3:** Mete-regression outcomes.

Moderator	Beta	Upper limit	Lower limit	Standard error	*P*-value
Mortality					
Baseline TMT thickness	−0.2171	−1.0250	0.5907	0.4122	0.59
Baseline NIHSS score	−0.0605	−0.1218	0.0009	0.0313	0.05
Good functional outcomes					
Baseline TMT thickness	−0.4065	−1.0340	0.2211	0.3202	0.20
Baseline NIHSS score	−0.0254	−0.0711	0.0202	0.0233	0.27

TMT, temporal muscle thickness; NIHSS, National Institutes of Health Stroke Scale.

Two studies reported data on post-stroke dysphagia. In the study of Sakai et al. (18), 70 patients were examined for the risk of dysphagia based on baseline TMT thickness. At discharge, 44.3% of patients had mild dysphagia while 28.6% had severe dysphagia based on the functional oral intake score. The median TMT values were found to be 4.70, 5.75, and 5.75 mm in the severe & mild dysphagia, and normal groups, respectively. A significant relationship between muscle volume decline and advancing dysphagia was noted by the authors. On linear regression analysis, TMT was a significant predictor of dysphagia at discharge (*p* = 0.036). Another study of 148 participants by Yang et al. (23) also examined the risk of dysphagia based on TMT. Dysphagia in their study was defined as retention of nasogastric tube at 4 and 12 weeks. On logistic regression analysis, TMT was significantly associated with dysphagia at 4 and 12 weeks (*p* < 0.001). The authors also assessed the diagnostic accuracy of TMT for predicting dysphagia. At the cut-off of 5.8 mm, TMT had a sensitivity of 89.04% and specificity of 94.67% for predicting dysphagia with area under the curve value of 0.941 (95% CI:0.89–0.97). At a cut-off of 4.7 mm, it had a sensitivity of 82.76% and specificity of 81.51% with an area under the curve value of 0.861 (95% CI: 0.80–0.91).

## Discussion

In the current meta-analysis, we investigated the relationship between TMT and outcomes after acute stroke. We were able to pool data on mortality and functional outcomes while data on dysphagia could only be analyzed descriptively. The cumulative analysis showed that patients with low TMT had a significantly higher risk of mortality as compared to those with higher TMT values. Secondly, there was a tendency for good functional outcomes with higher TMT but the results were not statistically significant. The effect size also demonstrated variability during sensitivity analysis indicating that the results were not robust. The same was also noted on descriptive analysis of the included articles with some studies demonstrating good functional outcomes with high TMT while others reporting no association between the two. Such results are indeed intriguing and could be related to baseline differences in the patient characteristics, severity of stroke, TMT cut-off, treatment, definition of outcomes, and other unaccounted confounders. In the study of Nozoe et al. (17), which noted no association between TMT and functional outcomes, the baseline NIHSS score was just 3 which may have contributed to the non-significant results. In the study of Ravera et al. (25), good functional outcomes were scored as mRS of 0–3 instead of mRS 0–2 which may have resulted in a non-significant association. Pertaining to dysphagia, both the included studies found a statistically significant association between low TMT and risk of dysphagia.

Defining sarcopenia and assessing its prevalence is crucial in managing critically ill elderly patients (8). This is especially important in stroke as stroke and sarcopenia share a bidirectional relationship. Sarcopenic adults have a higher risk of stroke and sarcopenia is associated with worse outcomes after stroke (5, 7). Moreover, the risk of sarcopenia also increases after stroke mainly due to inadequate physical activity and malnutrition (26). To allow incorporation into clinical practice, the method of quantifying sarcopenia should be tailored according to the patient setting. Muscle mass measured by anthropometry at the level of the lumbar vertebrae, and upper or lower limbs has been associated with functional outcomes after stroke (27). However, these measurements require abdominal or appendicular CT which may not be performed in an acute stroke setting. Furthermore, measuring grip strength or performing a walking test may be difficult in patients with neurological disorders. CT head is a routine investigation in a stroke or neurosurgery patient which allows precise measurements of the masticatory muscles. The TMT, therefore, emerges as a novel marker to measure skeletal muscle mass and indirectly sarcopenia. This is particularly important in context of very elderly since age may not be an accurate indicator of prognosis (6). TMT can be a novel marker in this specific subgroup which is vulnerable to both stroke and sarcopenia (5, 7).

A recent meta-analysis by Yang et al. (13) has shown that TMT can prognosticate patients with brain tumors. Low TMT was associated with poor overall survival and worse progression-free survival in both primary brain tumors and brain metastases. Similar results have been demonstrated even in head and neck cancer patients wherein TMT was found to be an independent predictor of progression-free survival (28). Another recent cohort study has noted that low TMT in subdural hematoma patients was associated with poor outcomes at discharge and reduced performance status at 3 months (29). Low TMT has been also linked with a higher risk of intracranial hemorrhage, poor functional outcomes, and a greater risk of falls in patients with traumatic brain injury (30). MRI-based TMT measurements have been associated with survival and functional outcomes in Amyotrophic Lateral Sclerosis (31). A longitudinal cohort study by Borda et al. (32) has shown that TMT had a significant association with cognitive performance and functional decline in Lewy body dementia. Moreover, TMT was a predictor of mortality in both Alzheimer’s disease and Lewy body dementia. However, there are differing results as well. Karadeg et al. (33) in a cohort study of 478 subarachnoid hemorrhage patients noted no significant relationship between TMT and functional outcomes at 6 months. The variability of results in some of the studies in our review as well as those in the literature indicate that while TMT could be a possible predictor of prognosis, there may be other variables at play, and more research is needed to establish TMT as a robust marker for predicting outcomes.

In support of TMT, there is research indicating a positive correlation with other skeletal muscle mass dimensions as well as functional measures. Park et al. (34) in a small study of 28 individuals have noted a moderate positive correlation between TMT and thigh & calf circumference as well as handgrip strength. However, they failed to demonstrate a correlation between TMT and physical performance. Another study shows a strong correlation between TMT and calf and arm muscle circumference (35). Steindl et al. (36) using a sample of 422 healthy individuals and 130 patients with neurological disorders have noted a strong significant correlation between TMT and grip strength suggesting that TMT should be used in the workup of all neurological patients. Likewise, TMT has been validated against EWGSOP1 and EWGSOP2 definitions of sarcopenia and found to strongly correlate with frailty status (37). Nevertheless, it must be noted that sarcopenia is an age-related loss of muscle mass and function which can vary throughout the body and with every individual. Muscle groups rich in type 2 (fast-twitch) fibers show early loss; like the anterior thigh muscles which are needed for mobility (38). There is also evidence that a differential association exists between site-specific muscle loss and functional outcomes after stroke (27).

Another factor hampering the use of TMT is a consensus on the optimal cut-off to define sarcopenia. As noted in the present review, different definitions were used based on analysis of the specific cohort data or using values from the literature. In this context, the recent study by Pesonen et al. (39) may help standardize TMT cut-offs. Using age and sex-adjusted data, the authors have reported a TMT cut-off of ≤ 4.09 mm and ≤3.44 mm from males and females, respectively, based on the EWGSOP criteria of sarcopenia. These values can be used in future research to determine the prognostic ability of TMT for all stroke outcomes. Such standardization would improve the quality of present evidence and allow the generalization of current evidence.

Muscle wasting is commonly noted in a critically ill patients (40, 41). Importantly, longitudinal changes in muscle volume may alter the association between TMT and post-stroke outcomes. Research shows that TMT volume decreases by 7.9% every week in a neurosurgical patient (40). Repeated assessments of the rectus femoris cross-sectional area have indicated a daily muscle volume reduction of around 2%. Consistently, repeated CT evaluations of quadriceps muscle volume indicated a daily reduction in muscle mass of 2.9% (41). The reduction in temporal muscle volume reported in literature is less pronounced than in these other muscles, likely due to the greater influence of critical illness polyneuropathy and critical illness myopathy on peripheral nerves and muscles (40). Hence, TMT may potentially be a more stable indicator in comparison with other peripheral muscles while assessing sarcopenia and its impact on outcomes. Currently, the relationship between alterations in temporal muscle volume and post-stroke outcomes remains ambiguous, necessitating further research in this area.

We need to acknowledge the limitations of our review. Scarce data and variability in reporting of outcomes meant that only a limited number of studies were available for each meta-analysis. We could not comprehensively assess the effect of confounders via a subgroup analysis due to limited data. At present, we cannot comment on how sex, stroke type, stroke severity, and treatment affect the association between TMT and outcomes. Specifically, sex-based differences in TMT thickness could be a major source of bias and may lead to variable cut-offs between men and women. However, sex-specific data was not available from any of the studies to permit a separate analysis. There was also much variability in the TMT cut-offs and follow-up intervals between the studies. Much of the data was short-term and the long-term impact of low TMT remains elusive. Presently, it also remains unclear on when does temporal muscle loss occur in sarcopenia patients. Late effects on temporal muscle may limit the applicability of TMT in early sarcopenia cases. The included studies also used only singular measurements of TMT. The quality of data was not high, most studies did not undertake propensity-score matching to eliminate the role of confounders. Lastly, moderate to substantial heterogeneity was noted in the meta-analysis which could be due to variations in the study populations. Hence, the results should be interpreted with caution.

Future prospective studies using standardized cut-offs of TMT assessing both short and long-term outcomes are needed to supplement the present evidence. Such studies should take into account variations in TMT thickness between men and women and present segregated data to assess sex-based difference in TMT and in turn effects on prognosis. Studies should also conduct multiple assessments of TMT to elucidate the effects of changes in muscle thickness over time and its association with outcomes. Lastly, all future studies should conduct a robust multivariate analysis including all possible confounders to present the best possible evidence.

## Conclusion

Preliminary evidence indicates that TMT could be a predictor of mortality but not functional outcomes after stroke. Low TMT scores also seem to be associated with the risk of dysphagia. The low-quality and scarce data precludes its routine use. Further robust research is needed to establish the role of TMT in assessing stroke prognosis.

## Data Availability

The original contributions presented in the study are included in the article material, further inquiries can be directed to the corresponding author.
